# Moving Forward to Disentangle the Role of the Brain in Human Obesity

**DOI:** 10.3390/brainsci14121245

**Published:** 2024-12-12

**Authors:** Swen Hesse

**Affiliations:** 1 Department of Nuclear Medicine, Leipzig University Medical Center, 04013 Leipzig, Germany; swen.hesse@medizin.uni-leipzig.de; 2 Integrated Research and Treatment Center Adiposity Diseases, Leipzig University Medical Center, 04103 Leipzig, Germany

Obesity and the associated medical sequelae pose a major challenge for healthcare systems worldwide. The standard approaches for treating obesity result in moderate weight loss, which, in most cases, cannot be maintained in the long term [1]. Therefore, the development of interventions that enable sustainable weight loss is of paramount importance. This requires a thorough investigation of the key biological and behavioral mechanisms that regulate energy balance.

This Special Issue of *Brain Sciences* highlights research on the role of the brain in the pathogenesis of obesity and how a disruption in the central regulation of eating behavior can lead to obesity. The goal of this Special Issue is to foster a deeper understanding of the relevant molecular and neural mechanisms to define the impact of brain sciences on the development of effective prevention and therapeutic strategies in human obesity. In addition, increasing our knowledge of the role of obesity in cognition should also be emphasized.

The neurotransmitters serotonin and noradrenaline have long been recognized as important targets in the treatment of obesity as drugs that act on these systems cause a change in body weight [2]. Advances in new molecular neuroimaging techniques have provided insights into the working of these and other neuroreceptors and their changes in vivo [3]. The study by Griebsch et al. uses positron emission tomography (PET) and highly selective radioligands to investigate mechanisms associated with weight loss for both the noradrenaline and serotonin transporters. The authors demonstrate that these transporters are involved in the pathomechanism of obesity in different ways, but both have the potential to serve as good predictors of treatment outcomes.

New and interesting pharmacological targets include the nicotinic acetylcholine receptors (nAChR), specifically the α4β2* nAChR [4], not least due to the observation that people who stop smoking gain a lot of weight in the long term [5]. Furthermore, according to preclinical data, individuals who have a propensity to attribute high-incentive salience to reward cues exhibit relatively poor attentional control mediated by α4β2* nAChR [6]. In a pilot study, Schweickert de Palma et al. investigated these receptors for the first time in vivo, comparing individuals with obesity and normal-weight controls. Their baseline findings indicate aberrant (i.e., lower) α4β2* nAChR availability in the key brain regions that regulate eating behavior.

Investigations on the cross-talk between central and peripheral factors will also contribute to the rich enhancement of our understanding of how targeting the brain is fundamental for achieving and maintaining a healthy body weight [7]. Schinke et al. showed that a dysregulation of the responsiveness of the stress axis in obesity is associated with serotonin signaling in the brain, particularly in limbic areas, and with changes in reward sensitivity.

The effects of obesity on the brain extend beyond energy regulation to cognitive health [8]. Large-scale epidemiological studies have shown that body mass index (BMI) and cognition are dynamically linked [9]. Studies also showed that high BMI correlates negatively with decision-making impairment and other cognitive parameters, but the nature of the relationship remains largely unexplored [10]. A prospective cohort study based on data of the UK biobank by Schaefer and colleagues identified potential nutritional risk factors for incident dementia. In another prospective study, Hansen et al. examined the relationships between preschoolers’ BMI and cognitive development. Their study revealed that excessive body mass may contribute to numerous biological, psychological, and social factors that inhibit children with obesity from reaching their full cognitive potential during a time in which brain development and cognitive skills development are at critical points of growth. Thus, early childhood obesity interventions may have positive consequences for cognitive development.

In general, patients with metabolic syndrome are often prone to cognitive decline [11]. According to the work of Gierach et al., this affects verbal fluency, suggesting executive dysfunction amongst people with metabolic syndrome. The authors assume that proper correction of the components of metabolic syndromes improves cognitive function. Executive dysfunction is also an intervention target in binge-eating disorder (BED) [12]. The findings on executive functions (EF) in BED, however, have thus far been inconsistent, and they are possibly biased by associated comorbidities. The study by Busch and colleagues sheds further light on the association of specific EF deficits in BED, warranting longitudinal studies to assess the importance of EF in the development of BED.

While α4β2* nAChR appears as a new pharmacological treatment target, glucagon-like peptide-1 (GLP-1) receptor agonists are currently used not only as anti-diabetic drugs but also for weight control worldwide [13]. A first study by Deden et al. on a specific GLP-1 PET ligand (^68^Ga-NODAGA-exendin-4) in people with obesity undergoing RYGB failed to achieve robust quantifiable uptake in regions of the brain beyond the hypothalamus. However, these radiotracers, in combination with other specific radioligands, for example, for the nicotinic system and the α4β2* nAChR, respectively, hold promise in further exploring the basic mechanism in the pathogenesis of obesity in humans in vivo [14].

Most PET studies published till now have addressed the brain dopamine system since this system is highly involved in the motivation to eat [15]. However, a study by van Son et al. did not find an association between food craving and striatal dopamine transporter availability, although the scores for food craving were higher in obesity. Accordingly, Janssen and Horstmann state in their comprehensive review on PET and single-photon emission computed tomography (SPECT) studies that based on the current data, it is unlikely that obesity can be traced back to a single dopaminergic cause or consequence.

Another important axis in the pathogenesis of obesity is the communication between the brain and the brown adipose tissue (BAT) [16]. A better understanding of the multifaceted communicative links between the brain and the BAT will open avenues for novel therapeutic approaches to treat obesity and related metabolic disorders, as has been shown, rather excellently, by Till and colleagues in their conceptual review.

Taken together, the regulation of food intake in humans is a complex process controlled by the dynamic interaction of homeostatic and hedonic systems, including homeostatic signalling from the gut, adipose tissue, and the vagus nerve, but also reward processes that orchestrate hedonic regulation. The review by Campos et al. focuses on the available neuroimaging evidence to describe this interaction between the homeostatic and hedonic components in human food intake regulation.

The understanding of the brain regulation of eating and energy balance has improved significantly over the last years. Currently, this knowledge is being translated into new treatments or interventions to mitigate obesity. However, more research is needed to unravel the complexity of brain–body interactions in order to reshape public health strategies and improve outcomes for millions of people with obesity worldwide. We hope that this Special Issue can provide readers with an insight into this exciting field of research, and that it might also provide inspiration for future research.
